# Identification and Functional Characterization of Alternative Transcripts of LncRNA HNF1A-AS1 and Their Impacts on Cell Growth, Differentiation, Liver Diseases, and in Response to Drug Induction

**DOI:** 10.3390/ncrna10020028

**Published:** 2024-04-21

**Authors:** Jing Jin, Le Tra Giang Nguyen, Andrew Wassef, Ragui Sadek, Timothy M. Schmitt, Grace L. Guo, Theodore P. Rasmussen, Xiao-bo Zhong

**Affiliations:** 1 Department of Pharmaceutical Sciences, School of Pharmacy, University of Connecticut, Storrs, CT 06269, USA; jingjin.w@uconn.edu (J.J.); le_tra_giang.nguyen@uconn.edu (L.T.G.N.); theodore.rasmussen@uconn.edu (T.P.R.); 2 Department of Pharmaceutics, Ernest Mario School of Pharmacy, Rutgers University, Piscataway, NJ 08901, USA; awassef@scarletmail.rutgers.edu; 3 Center of Excellence for Pharmaceutical Translational Research and Education, Rutgers University, Piscataway, NJ 08901, USA; 4 Center of Excellence for Metabolic and Bariatric Surgery, Robert Wood Johnson Barnabas University Hospital, New Brunswick, NJ 08901, USA; raguis@advancedsab.com; 5 Department of General Surgery, University of Kansas Medical Center, Kansas City, KS 66160, USA; tschmitt@kumc.edu; 6 Department of Pharmacology and Toxicology, Ernst Mario School of Pharmacy, Rutgers University, Piscataway, NJ 08901, USA; guo@eohsi.rutgers.edu

**Keywords:** alternative transcript, CYP3A4, HepaRG, HNF1A-AS1

## Abstract

The long non-coding RNA (lncRNA) hepatocyte nuclear factor-1 alpha (HNF1A) antisense RNA 1 (HNF1A-AS1) is an important lncRNA for liver growth, development, cell differentiation, and drug metabolism. Like many lncRNAs, HNF1A-AS1 has multiple annotated alternative transcripts in the human genome. Several fundamental biological questions are still not solved: (1) How many transcripts really exist in biological samples, such as liver samples and liver cell lines? (2) What are the expression patterns of different alternative HNF1A-AS1 transcripts at different conditions, including during cell growth and development, after exposure to xenobiotics (such as drugs), and in disease conditions, such as metabolic dysfunction-associated steatotic liver disease (MASLD), alcohol-associated liver disease (ALD) cirrhosis, and obesity? (3) Does the siRNA used in previous studies knock down one or multiple transcripts? (4) Do different transcripts have the same or different functions for gene regulation? The presented data confirm the existence of several annotated HNF1A-AS1 transcripts in liver samples and cell lines, but also identify some new transcripts, which are not annotated in the Ensembl genome database. Expression patterns of the identified HNF1A-AS1 transcripts are highly correlated with the cell differentiation of matured hepatocyte-like cells from human embryonic stem cells (hESC), growth and differentiation of HepaRG cells, in response to rifampicin induction, and in various liver disease conditions. The expression levels of the HNF1A-AS1 transcripts are also highly correlated to the expression of cytochrome P450 enzymes, such as CYP3A4, during HepaRG growth, differentiation, and in response to rifampicin induction.

## 1. Introduction

The long non-coding RNA (lncRNA) hepatocyte nuclear factor-1 alpha (HNF1A) antisense RNA 1 (HNF1A-AS1) is an important lncRNA for liver growth, development, cell differentiation, and cellular functions, such as drug metabolism. HNF1A-AS1 was first reported to be abnormally upregulated in esophageal tumorigenesis [1] and continuingly reported to be involved in proliferation and metastasis in various carcinomas, including lung adenocarcinoma [2,3], gastric adenocarcinoma [4,5], hepatocellular carcinoma [6,7], colorectal carcinoma [8,9], and other cancers [10,11]. HNF1A-AS1 has also been shown to be involved in an HNF1A mediated transcriptional regulatory network to control the basal and drug-induced expression of drug metabolizing cytochrome P450 (CYP) enzymes in liver cells [12,13]. As a CYP regulator, HNF1A-AS1 can alter susceptibility of cytotoxicity in liver cells and is induced by various drugs, such as acetaminophen [14] and ritonavir [15], which are primarily metabolized by certain CYP enzymes. HNF1A-AS1 regulates CYP expression at both transcription and post-transcription levels. At the transcriptional level, HNF1A-AS1 serves as an RNA scaffold to interact with nuclear receptors, such as pregnane X receptor (PXR), and histone modification enzymes, such as arginine methyltransferase 1, to alter histone modifications of the *CYP3A4* gene as a component of its transcriptional regulation [16]. At the post-transcriptional level, HNF1A-AS1 interacts with HNF1A protein to block its interaction with the E3 ubiquitin ligase tripartite motif containing 25 (TRIM25) for preventing HNF1A ubiquitination and protein degradation, further regulating the expression of CYP3A4 [16]. These studies demonstrate that HNF1A-AS1 is a critical lncRNA involved in the regulation of liver growth, differentiation, and metabolic functions.

Individual alternative transcripts of HNF1A-AS1 have not been assessed for their functions. The remarkable complexity and diversity of cellular and organismal functions in eukaryotes cannot be solely attributed to the sheer number of genes in their genomes. Instead, it primarily arises from the intricate orchestration of gene expression, resulting in the generation of multiple transcripts and proteins from the same genes. This phenomenon is known as transcript diversification [17,18,19,20,21,22,23]. In the Ensembl genome browser (GRCh38.p14), there are currently 19,830 annotated coding genes and 26,462 annotated noncoding genes. Interestingly, these genes give rise to a much larger number of transcripts, estimated to be 252,989, with an average of around 5.5 transcripts per gene, indicating multiple transcripts are a common phenomenon. Alternative promoter usage and alternative splicing are two major processes driving transcript diversification at the RNA level [19,20]. Alternative transcription involves the differential utilization of transcription start sites, which is influenced by factors, such as regulatory elements, chromatin structure, and transcription factor binding, resulting in the generation of multiple pre-RNAs [24,25,26]. Subsequently, pre-RNAs undergo alternative splicing, where different combinations of exons are joined together, leading to the production of RNA transcripts with distinct sequences. The regulation of alternative splicing is mediated by splicing factors and regulatory elements, including splice enhancers and silencers, which govern the selection of splice sites and exon inclusion/exclusion decisions [27,28]. Both alternative transcription and splicing processes can be tissue-specific, temporally regulated, or influenced by environmental cues, enabling cells to adapt to different contexts [29].

Alternative promoter usage and alternative splicing mechanisms exhibit dynamic variations across different tissues and developmental stages and are frequently disrupted in human diseases [30]. This phenomenon has been observed in numerous cancer types, where abnormal splicing patterns in tumor tissues have been identified using powerful high-throughput sequencing technologies [31,32,33]. Moreover, investigations into the functional roles of non-coding RNAs (ncRNAs) have unveiled many distinct functions, including roles in alternative splicing events [34]. LncRNAs have garnered significant interest due to their diverse functions. Following alternative splicing events, pre-lncRNAs can give rise to distinct mature lncRNA transcripts that may possess either similar or divergent biological functions. A compelling example is that of lncRNA-PXN-AS1, where alternative splicing generates transcripts with and without exon 4. Interestingly, the transcript containing exon 4 promotes liver cancer, while the transcript lacking exon 4 inhibits liver cancer progression [35]. Another notable example involves the pre-lncRNA PVT1, which gives rise to two distinct mature lncRNA transcripts. Both transcripts have been implicated in promoting tumor growth in renal cell carcinoma [36]. Therefore, the identification and characterization of alternative RNA transcripts are crucial for understanding the mechanisms in many human diseases, including cancer [30,31].

LncRNA HNF1A-AS1 is encoded by the *HNF1A-AS1* gene, which is located on the negative strand in the antisense direction relative to the *HNF1A* gene with a genomic locus spanning nucleotides 120,941,728 to 120,980,968 on chromosome 12 in the Ensembl genome browser based on the Human (GRCH38) database. As a typical lncRNA gene, *HNF1A-AS1* has multiple alternative transcripts annotated by both Ensembl genome browser and UCSC genome browser, including HNF1A-AS1-201, 202, 203, 204, 205, 206, 207, 208, and 209 produced by either alternative transcription or alternative splicing. Several fundamental questions for the transcript diversity of HNF1A-AS1 are still not answered, including: (1) How many transcripts really exist in biological samples, such as liver samples and liver cell lines? (2) What are the expression patterns of different alternative HNF1A-AS1 transcripts at different conditions, including during cell growth and development, after exposure to xenobiotics (such as drugs), and in disease conditions, such as metabolic dysfunction-associated steatotic liver disease (MASLD), alcohol-associated liver disease (ALD) cirrhosis, and obesity? (3) Does the siRNA used in previous studies knock down one or multiple transcripts? (4) Do different transcripts have the same or different functions for gene regulation? This study aims to explore these questions.

## 2. Results

### 2.1. Identification of HNF1A-AS1 Alternative Transcripts in Human Livers and Cultured Cells

**Figure 1:**
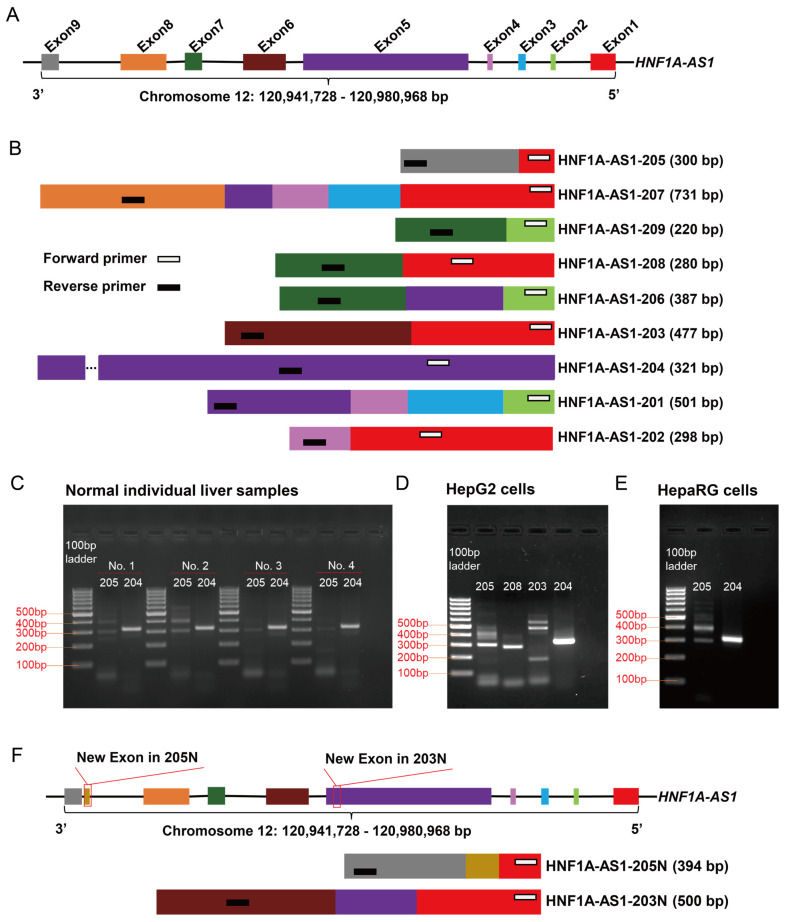
Identification of HNF1A-AS1 transcripts in human liver samples and liver cell lines. (**A**) The genomic organization of the *HNF1A-AS1* gene with nine exons from 12: 120,941,728–120,980,968 bp on chromosome 12. (**B**) Nine annotated HNF1A-AS1 transcripts after alternative transcription and alternative splicing are shown in the figure with color rectangles representing the relative lengths of each transcript. The forward primers (white bars) and reverse primers (black bars) were designed to amplify from the first exon to the last exon for each transcript. The distances between forward primers and reverse primers represent the relative lengths of the PCR products. However, primer lengths are not scaled. (**C**) In the four normal individual human liver samples, three transcripts of HNF1A-AS1 were confirmed with two annotated (205 and 204) and one new (205N). (**D**) In HepG2 cells, a total of six transcripts of HNF1A-AS1 with four annotated (205, 208, 203, and 204) and two new (205N and 203N) were confirmed. (**E**) In HepaRG cells, three transcripts of HNF1A-AS1 with two annotated (205 and 204) and one new (205N) were confirmed. (**F**) 205N contains a 94 bp exon (retaining intron between exon 8 and exon 9). 203N represents a new transcript with a 73 bp exon (part of exon 5). The sequences of all confirmed and new identified transcripts are listed in Supplemental Table S1.

*HNF1A-AS1* gene is located at chromosome 12 from 120,941,728 to 120,980,968 bp in the negative strand with nine identified exons (Figure 1A). Nine alternative HNF1A-AS1 transcripts have been annotated, and these range from 343 bps (HNF1A-AS1-205) to 2,455 bps (HNF1A-AS1-204) in the Ensembl genome browser based on the Human (GRCH38.p14) build, with detailed information shown in Table 1. Alternative transcription and alternative splicing contribute to the diversity of the nine annotated transcripts of HNF1A-AS1 (Figure 1B). Alternative transcripts of HNF1A-AS1-201 to HNF1A-AS1-209 will be represented by transcript 201–209 in the following text. Transcript 204 is derived from a single exon, Exon 5. Other transcripts contain two or more exons (up to 5 for 207). Some pairs of transcripts may not share any sequence, for example 204 and 205, implying that they may have different functions.

Though the existing information on transcript diversity is useful, it should be noted that the annotated alternative transcripts of HNF1A-AS1 are based solely on the analysis of RNA-Seq data from various biological samples, which have not been validated by experiments. To determine the actual number of transcripts presented in human liver samples and liver cell lines, primers for polymerase chain rection (PCR) were designed to amplify each transcript. Forward primers were selected from the first exons (an open bar in Figure 1B), while the reverse primers were chosen from the last exons (a solid black bar in Figure 1B). The sizes of their amplification products are shown in Figure 1B and Table 2. In four individual human liver samples, two bands were observed for 205, while one band was observed for 204 (Figure 1C). Subsequently, all the bands were purified for Sanger sequencing. The sequence results reveal that the upper band for 205 corresponds to a new transcript called HNF1A-AS1-205N (205N), which contains a 94 bp exon (retaining intron between exon 8 and exon 9) (Figure 1F). The lower band for 205 and the band for 204 were found to match the annotations in the genome browser (Figure 1C). Notably, the expression levels of 205N and 205 were similar in liver samples No.1 and No.2, but the expression levels of 205N were lower than 205 in the liver samples of No.3 and No.4. Interindividual variations may account for this observed phenotype. In comparison to the results of the liver samples, the results of the liver cell lines yielded different findings. In HepG2 cells, a total of seven bands were detected, including two bands for 205, one band for 208, three bands for 203, and one band for 204 (Figure 1D). Sanger sequencing confirmed that the upper band for 205 corresponds to 205N, the upper band for 203 represents a new transcript with a 73 bp exon (part of exon 5) (Figure 1F), and the lower band for 203 was a mismatch. The remaining bands were consistent with the annotated transcripts of 205, 208, 203, and 204, respectively. Furthermore, HepaRG cells exhibited the same band patterns as observed in the human liver samples, with the only difference being that the expression level of 205N was more than that of 205 (Figure 1E). All the sequence information of the identified transcripts is listed in Supplemental Table S1.

### 2.2. Design of Real-Time Quantitative PCR (RT-qPCR) Primers for Quantification of the Identified Transcripts

**Figure 2:**
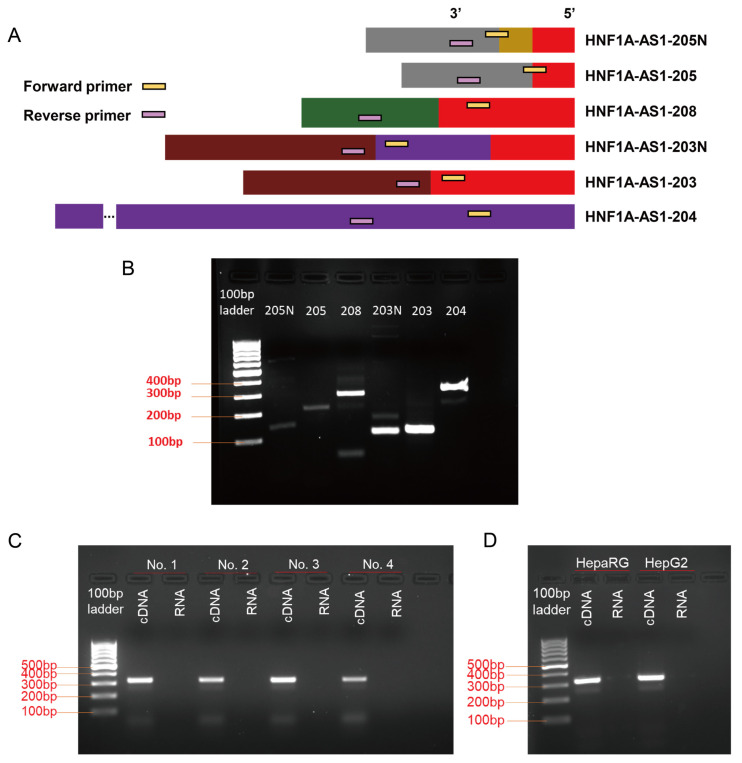
Design of RT-qPCR primers for quantification of the identified transcripts. (**A**) Schematic representation of the PCR primer locations designed for each transcript, ensuring specific quantification of the individual transcripts. (**B**) The PCR results demonstrated the amplification of either a single band or amplification of the majority of the specific transcript using the respective RT-qPCR primer pairs. (**C**) PCR amplification of HNF1A-AS1-204 in normal individual liver samples using cDNA or RNA as templates. There was no amplification in lanes using RNA as a template, indicating no detectable genomic DNA contamination. (**D**) PCR amplification of 204 in HepaRG and HepG2 cells using cDNA or RNA as templates. The faint bands were observed in lanes using RNA as a template, indicating minor genomic DNA contamination.

RT-qPCR was used to measure expression levels of the alternative transcripts under different conditions, using appropriately designed RT-qPCR primer pairs. In this study, we designed specific primers for 205N, 208, 203N, and 204, targeting adjacent exons. To distinguish between 205N and 205, and between 203N and 203, the forward primers for 205 and 203 were selected at the exon junctions (Figure 2A and Table 3). Gel electrophoresis analysis of the amplification products revealed the presence of either a single band or amplification of the majority of the specific transcripts in all the lanes, indicating the suitability of these primers for subsequent RT-qPCR quantification (Figure 2B).

However, designing primers for the transcript 204 presented a unique challenge as it comprises only one exon, raising potential concerns about genomic DNA contamination influencing the qPCR results. To address this, we performed PCR amplifications for the HNF1A-AS1-204 using both cDNA and RNA templates from individual normal liver samples, as well as HepaRG and HepG2 cell lines. As demonstrated in individual normal liver samples (Figure 2C), no amplification occurred in lanes using RNA as a template, indicating no detectable genomic DNA contamination. When it came to HepaRG and HepG2 cell samples (Figure 2D), amplification from cDNA templates produced clear bands for 204, indicating robust expression. Conversely, when RNA templates were used, only faint bands were observed, suggesting minor genomic DNA contamination. Furthermore, to quantitatively assess the impact of this contamination, we conducted qPCR analyses using RNA templates from individual normal livers, HepaRG, and HepG2 cells. The Cq values were undetectable on the machine (C1000 TouchTM Thermal Cycler with CFX96TM Real-Time System), strongly suggesting that the minor genomic DNA contamination in our cell samples will not influence the subsequent qPCR results.

### 2.3. Expression Patterns of HNF1A-AS1 Transcripts during Stages of Hepatocyte Differentiation from Human Embryonic Stem Cells (hESC)

**Figure 3:**
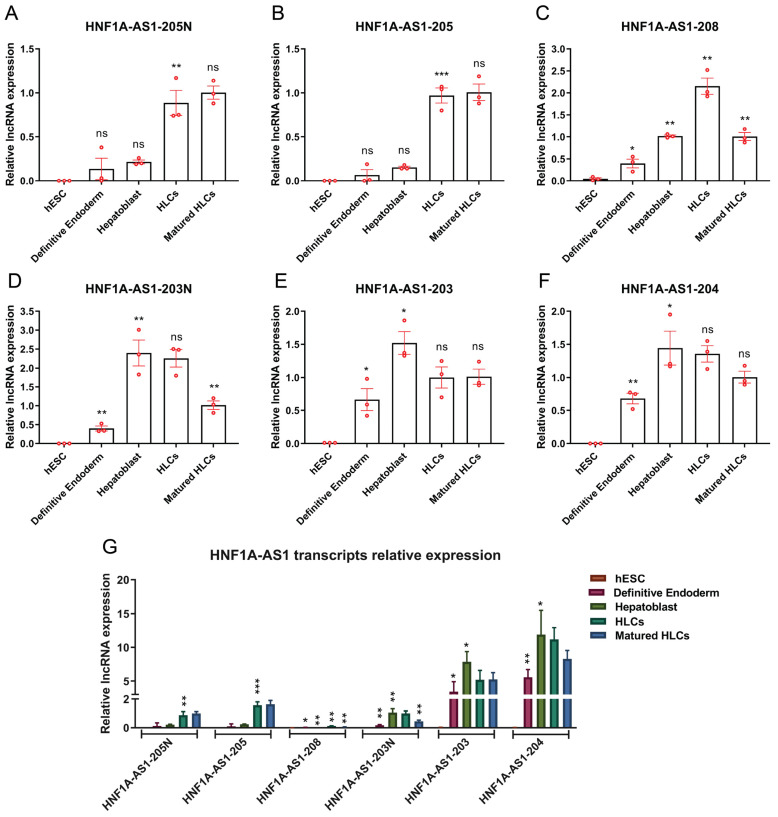
Expression patterns of the HNF1A-AS1 transcripts during different stages of matured hepatocyte-like cells’ differentiation from human embryonic stem cells (hESC). (**A**–**F**) Quantification of different HNF1A-AS1 transcripts of 205N (**A**), 205 (**B**), 208 (**C**), 203N (**D**), 203 (**E**), and 204 (**F**) at different stages of hepatocyte differentiation from hESC using RT-qPCR. (**G**) Relative expression levels of all transcripts of HNF1A-AS1 at different stages from hESC to hepatocytes measured by RT-qPCR. The data are presented as mean ± standard deviation (SD) (n = 3). Statistical analysis was conducted using a *t*-test, comparing each column to its previous column. * *p* < 0.05; ** *p* < 0.01; *** *p* < 0.001; ns: no significance.

Understanding the dynamics of transcript expression during the formation of hepatocytes is crucial before delving into a more comprehensive exploration of their functions in drug metabolism in liver cells. In this study, we induced human embryonic stem cells to differentiate into definitive endoderm, followed by hepatoblast cells, hepatocyte-like cells (HLCs), and finally matured HLCs. Throughout these five stages, the expression profiles of transcripts 205N (Figure 3A) and 205 (Figure 3B) were examined, which exhibited a significant increase in expression specifically during the HLCs stage, and maintained this elevated expression level in the subsequent matured HLCs stage. On the other hand, transcripts 208 (Figure 3C), 203N (Figure 3D), 203 (Figure 3E), and 204 (Figure 3F) became strongly expressed during the definitive endoderm stage. Notably, transcripts 208 and 203N exhibited an initial increase but subsequently decreased in the matured HLCs stage. In contrast, transcripts 203 and 204 displayed an initial increase in expression and maintained a consistently high level throughout the following stages. In comparison to the relative expression levels of all transcripts, it was evident that transcripts 203 and 204 exhibited relatively higher expression levels compared to the other transcripts (Figure 3G).

### 2.4. Expression Patterns of the Identified HNF1A-AS1 Transcripts during HepaRG Cell Growth and Differentiation

**Figure 4:**
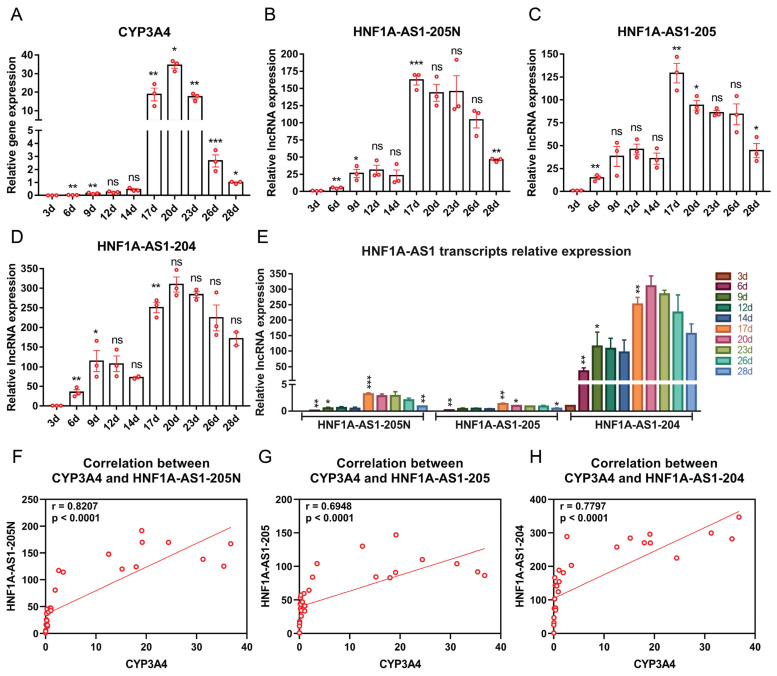
Expression patterns of the identified HNF1A-AS1 transcripts during HepaRG cell growth and differentiation. (**A**) CYP3A4 mRNA levels at different stages of HepaRG cells from the growth (3–14 d) to the differentiation (17–28 d) period measured by RT-qPCR. (**B**–**D**) Relative RNA expression levels of different transcripts of HNF1A-AS1 at different stages of HepaRG cells from the growth to the differentiation period measured by RT-qPCR. (**E**) Relative expression levels of all transcripts of HNF1A-AS1 at different stages from the growth to the differentiation period measured by RT-qPCR. Statistical analysis was performed using a *t*-test, comparing each column to the previous column. * *p* < 0.05; ** *p* < 0.01; *** *p* < 0.001; ns: no significance. (**F**–**H**) Pearson correlation analyses between different HNF1A-AS1 transcripts and CYP3A4 expression levels.

The HepaRG cell line is widely regarded as an appropriate model for studying drug metabolism due to its abundant expression of Cytochrome P450 (CYP) mRNAs and proteins. HepaRG expand without differentiation for two weeks (growth phase), followed by an additional two weeks for differentiation to reach a sufficiently mature state for experimentation. In this study, we focused on CYP3A4, a representative CYP member, and measured its expression levels at various stages of HepaRG cell growth and differentiation using RT-qPCR. Interestingly, during the growth phase, CYP3A4 expression remained relatively low. However, a significant increase was observed during the differentiation period, indicating a correlation between CYP3A4 expression and the maturation of HepaRG cells (Figure 4A). To gain further insights, we also measured the expression levels of transcripts 205N (Figure 4B), 205 (Figure 4C), and 204 (Figure 4D) at the same time points. Notably, these transcripts displayed similar expression kinetics as CYP3A4, with relatively low expression during the growth phase and significantly higher expression during the differentiation period. In comparison to the relative expression levels of all the transcripts, it was evident that transcript 204 exhibited higher expression levels compared to the other transcripts (Figure 4E). To explore the relationship between these transcripts and CYP3A4 during HepaRG maturation, we conducted a Pearson correlation analysis (Figure 4F–H). Remarkably, all three transcripts exhibited a strong positive correlation with CYP3A4.

### 2.5. Expression Patterns of HNF1A-AS1 Transcripts during Rifampicin Treatment

**Figure 5:**
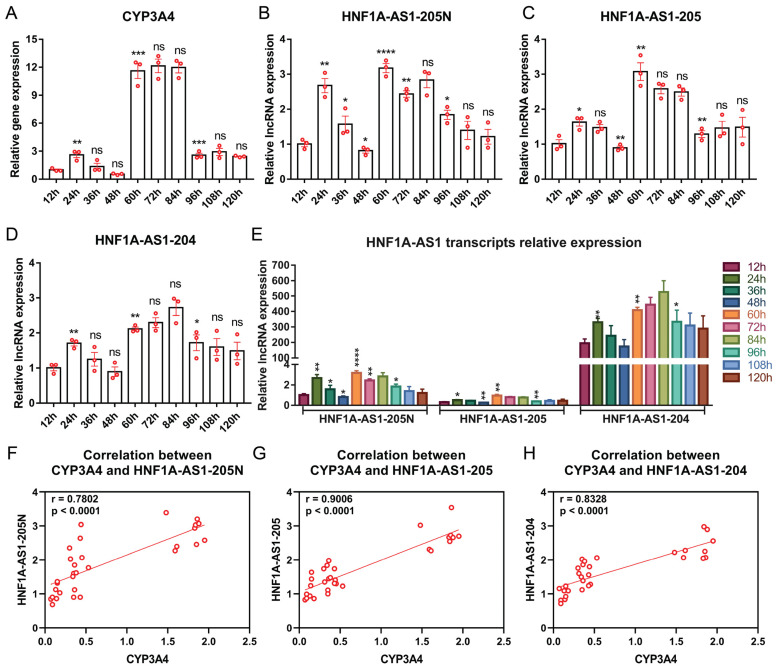
Expression patterns of the identified HNF1A-AS1 transcripts during rifampicin treatment. (**A**) CYP3A4 expression levels after rifampicin treatment measured at 12-h intervals. (**B**–**D**) Expression levels of different transcripts of HNF1A-AS1 after rifampicin treatment measured at 12-h intervals. (**E**) Relative expression levels of the HNF1A-AS1 transcripts at 12-h intervals following rifampicin treatment, measured by RT-qPCR. Statistical analysis was conducted using *t*-tests. * *p* < 0.05; ** *p* < 0.01; *** *p* < 0.001; **** *p* < 0.0001; ns: no significance. (**F**–**H**) Pearson correlation analyses of expression levels between different HNF1A-AS1 transcripts and CYP3A4.

Rifampicin is a well-known inducer that increases the expression of CYP3A4 after treatment. After the maturation of HepaRG cells at twenty-eight days, a treatment of 10 nmol rifampicin was administered for 120 h. Throughout this period, CYP3A4 expression levels were measured every 12 h using RT-qPCR. The results reveal that during the initial 48 h, CYP3A4 expression levels remained relatively low. However, from 60 h onwards, there was a significant increase in CYP3A4 expression, reaching a plateau until 84 h, after which it decreased back to a similar level as that observed from 12 to 48 h (Figure 5A). To compare the expression profiles of the HNF1A-AS1 transcripts with CYP3A4, their expression levels were measured at the same time points. Notably, all three transcripts displayed similar expression patterns to CYP3A4 (Figure 5B–D). Furthermore, when comparing the expression levels of all the transcripts together, it was observed that transcript 204 exhibited the highest level of expression (Figure 5E). To analyze the relationship between CYP3A4 and these three transcripts during the drug induction process, Pearson correlation analyses were conducted. The results demonstrate strong correlations between all the transcripts and CYP3A4 (Figure 5F–H).

### 2.6. Expression Patterns of the Identified HNF1A-AS1 Transcripts in Different Liver Disease Conditions

**Figure 6:**
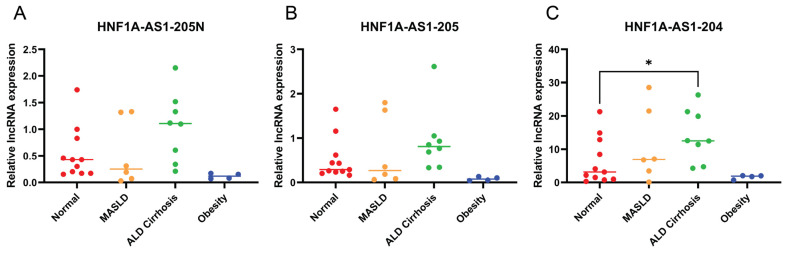
Expression patterns of the identified HNF1A-AS1 transcripts in different liver disease conditions. (**A**–**C**) Expression levels of the HNF1A-AS1 transcripts in four distinct groups: normal livers, metabolic dysfunction-associated steatotic liver disease (MASLD), alcohol-associated liver disease (ALD) cirrhosis, and liver samples from obesity individuals (BMI > 35). Each group consisted of four to eleven samples. Statistical analysis was performed using t-tests to compare the expression levels between the disease groups and the normal group. * *p* < 0.05.

Analyzing the expression levels of the HNF1A-AS1 transcripts in various disease conditions could provide valuable insights into their functional roles, disease associations, and pharmacological implications. To investigate further, a comprehensive set of liver samples was collected, including eleven normal livers, six cases of MASLD, eight cases of ALD cirrhosis, and four livers from obese individuals with body mass index (BMI) > 35. The expression levels of the transcripts 205N, 205, and 204 were assessed in these liver samples. The results reveal an interesting finding: among the tested transcripts, only 204 demonstrated a significant increase specifically in cases of ALD cirrhosis (Figure 6C). At the same time, the other transcripts exhibited elevated expression levels in the same disease condition; however, the differences did not reach statistical significance (Figure 6A,B).

## 3. Discussion

The current study has addressed the following fundamental biological questions for alternative transcripts of HNF1A-AS1.

Question One: How many transcripts really exist in biological samples, such as liver samples and liver cell lines? Using specific designed PCR primers for each annotated transcript, the expressions of transcripts 204 and 205 were confirmed in the human liver samples and HepaRG cells, whereas the expressions of 203, 204, 205, and 208 were validated in HepG2 cells (Figure 1). In addition, two new transcripts of 203N and 205N were identified, which are not listed in the annotated database. Whether the alternative HNF1A-AS1 transcripts are tissue-specific needs to be investigated in future studies to derive a transcription profile of all transcripts in various tissues.

It is important to note that these findings indicate variations in the expression patterns of the identified transcripts among the different individual liver samples (sample 1-4) and liver cell lines (HepG2 vs. HepaRG). The results obtained from the Sanger sequencing provide valuable insights into the existence and characteristics of these transcripts, shedding light on the complexity of gene expression in human livers and liver cell lines. Further investigations are warranted to fully understand the functional implications of these variations and their potential contributions to liver biology.

Question Two: What are the expression patterns of different alternative HNF1A-AS1 transcripts at different conditions, including during cell growth and development, after exposure to xenobiotics (such as drugs), and in liver disease conditions (such as MASLD, ALD cirrhosis, and obesity liver)?

Under certain culture conditions, hESC cells are able to generate hESC-derived matured hepatocyte-like cells via multiple phases as they differentiate into definitive endoderm, hepatoblasts, hepatocyte-like cells, and matured hepatocyte-like cells [37,38]. All alternative HNF1A-AS1 transcripts identified in the human liver samples and liver cell lines are expressed only at very low levels in hESC cells, if at all. Expression levels of nearly all transcripts are first induced in definitive endoderm, and further increased in hepatoblast- and hepatocyte-like, cells (Figure 3). Transcripts 204 and 203 are relatively more abundant than other transcripts during the differentiation process. These findings provide valuable insights into the dynamic expression patterns of these transcripts during hepatocyte development, and suggest potential developmental roles worthy of further investigation.

HepaRG cells, a proliferative human hepatoma-derived cell line, have the ability to differentiate into hepatocyte-like and biliary-like cells after being cultured in the growth phase for two weeks (day 1–14) followed by the differentiation phase for an additional two weeks (day 15–28) [39]. HepaRG cells maintain significantly higher expression levels of metabolism functions, including drug transporters and metabolizing enzymes, such as CYP3A4 (shown in Figure 4A), in the differentiation phase than the growth phase.

Transcripts 204, 205, and 205N all express significantly higher levels at the differentiated stage from day 17 to day 28 compared to the growth stage from day 1 to 14 (Figure 4B–D). Their expression levels are also significantly correlated with CYP3A4 levels throughout the entire growth and differentiation period (Figure 3F–H), suggesting their potential involvement in the alteration of CYP expression during HepaRG growth and differentiation. These findings imply that transcripts 204, 205, and 205N may play crucial roles in modulating CYP activity during HepaRG cell maturation.

Induction of expression of drug metabolizing enzymes in liver cells by numerous drugs is the major mechanism causing drug–drug interactions (DDIs) in clinical practice [40]. Rifampicin is a well-known CYP inducer causing DDIs, which involves the activation of nuclear receptors, such as PXR [41]. A significant increase in CYP3A4 expression is observed after the treatment of HepaRG cells with rifampicin for 60 h (Figure 5A). Transcripts 204, 205, and 205N also show a significant increase in expression at 60 h in comparison to 48 h in HepaRG cells (Figure 5B–D). For 120 h after the treatment of rifampicin in HepaRG cells, expression levels of CYP3A4 are highly correlated with 204, 205, and 205N in a Pearson correlation analysis (Figure 5F–H), indicating their potential functional involvement during the rifampicin induction process. These findings suggest that these transcripts may play important roles in modulating CYP3A4 expression and function during drug treatment.

HNF1A-AS1 was first identified as the most upregulated lncRNA in esophageal tumorigenesis, indicating its potential association with diseases [1]. This study examined relative expression levels of the alternative transcripts 204, 205, and 205N (Figure 6) in normal, MASLD, ALD cirrhosis, and obesity liver samples. Although the sample sizes are limited for each group, a significantly higher level of expression of 204 is identified in the ALD cirrhosis groups in comparison to the normal group. This observation suggests that transcript 204 may play a distinctive role in the context of ALD cirrhosis, potentially indicating its involvement in the disease pathogenesis or response to alcohol-induced liver damage. Future studies with bigger sample sizes for more disease conditions are needed to evaluate the role of HNF1A-AS1 in disease progress. HNF1A-AS1 has been suggested as a tumor-associated lncRNA [42] and has potential medical implications in cancer prevention and treatment [43].

Question Three: Do the siRNAs used in previous studies knock down one or multiple transcripts? siRNAs and shRNAs are the main approaches for knocking down HNF1A-AS1 in various in vitro systems to investigate its functions across different biological processes. Among the transcripts, HNF1A-AS1-204 is the most frequently studied. The siRNAs reported in the literature specifically target 204 [1,9,12,13,44]. However, the shRNA used in a study of Ritonavir-induced hepatoma cells targets multiple transcripts of HNF1A-AS1, specifically 204 and 206 [15]. It is important to note that not all publications provided the shRNA targeting sequences, which means the specific transcripts targeted remain unidentified [12,14,45]. In addition, the specificity of the primers used in all studies also requires careful consideration. While most papers only focused on the expression level of 204 [1,9,12,44], the primers used in some studies target two transcripts, 204 and 201 [13,15,16]. All the issues highlighted here may pose challenges for researchers seeking to repeat and compare the conclusions drawn in the publications. To address this, future studies should consistently provide clear information about the transcript IDs, siRNA and shRNA targeting sequences, and corresponding primers to enhance the reliability and reproducibility of research findings.

Question Four: Do different HNF1A-AS1 transcripts have the same or different functions related to the regulation of gene expression of CYPs? Although encoded by the *HNF1A-AS1* gene, the transcript of 204 has completed different sequences without any overlap with 203, 205, and 205N. Whether they have the same or different functions for gene regulation needs to be determined in future studies. Further experiments, such as knockdown and overexpression experiments targeting each transcript individually, should be performed in the future to gain a deeper understanding of their specific functions and mechanisms of action. Their subcellular locations in either nuclei or cytoplasm should be determined in different cells and different conditions.

In conclusion, the lncRNA *HNF1A-AS1* gene can transcribe multiple alternative transcripts in different tissues and cells by alternative transcription and splicing. Some transcripts have been annotated in the human genome, but not all have been experimentally determined. The transcripts expressed in hepatocyte cells, including 204, 205, and 205N, have dynamic expression patterns during hepatocyte differentiation from hESC cells, in the cell growth and differentiation of HepaRG cells, and in the induction of gene expression in HepaRG cells by xenobiotics, such as drugs. The dynamic expression patterns are also highly correlated with the expression patterns of CYP enzymes, such as CYP3A4. The previous studies on the functions of HNF1A-AS1 using siRNA and shRNA knockdown approaches only knocked down a particular transcript, but no other alternative transcripts. The identified HNF1A-AS1 transcripts have distinguished RNA sequences without any overlap, indicating that different HNF1A-AS1 transcripts may have different functions in the regulation of the expression of their target genes, which need to be determined in future studies.

## 4. Materials and Methods

### 4.1. Human Liver Samples Collection

Human liver RNA samples from seven normal individuals, six MASLD patients, and eight ALD cirrhosis patients were provided by the University of Kansas Liver Tissue Biorepository supported by grant 1P20GM144269-01 from the National Institute of General Medical Sciences. The samples were obtained in accordance with ethical guidelines and with appropriate informed consent.

In addition, human liver RNA samples from four normal individuals and four obesity patients were provided by the Department of Pharmacology and Toxicology of Rutgers University. Four obesity patients (BMI > 35), aged 18 to 80 years, undergoing bariatric surgery, required laparoscopic liver biopsy for suspected chronic liver disease. Liver biopsies, obtained via the wedge or needle core technique from the left liver lobe, were divided. A portion underwent standard pathological diagnosis, while the rest was allocated for research. Collected biopsies were promptly placed on ice, transported, and flash-frozen within 15 min to preserve the cellular and molecular integrity of the specimen. Four control liver specimens (BMI < 30) were sourced from established channels: the Cooperative Human Tissue Network (CHTN) and the National Disease Research Interchange (NDRI) (links to their respective websites: https://www.chtn.org/about/index.html and https://ndriresource.org/about-us; access date both on 12 January 2023). CHTN, backed by the National Cancer Institute, furnished human biospecimens and fluids from routine medical procedures for research purposes. NDRI, a nonprofit, provided human tissues from diverse healthy and afflicted donors. A standardized dataset, encompassing demographic details (age, race, gender), alongside tissue diagnosis quality control, was obtained from CHTN and NDRI. Accompanying the samples were deidentified pathology reports, following established confidentiality norms. The use of these de-identified human specimens was determined as exempt research by the Rutgers Biomedical Health Sciences Institutional Review Board (Pro2019001020). A total of 8 subjects were included. After exclusions, the study retained 8 participants. Exclusions were made for specimens from subjects with infectious diseases, a history of alcohol abuse/addiction, hepatitis A, B, or C infection (self-reported/medical records), and poor RNA quality. Ethical compliance was ensured via approval from the RBHS institutional review board under protocols Pro2020002744 and Pro2019001020, safeguarding ethical guidelines and participant welfare.

### 4.2. Differentiation of hESC to Hepatocytes In Vitro

ESCs were grown in mTeSR1 (#85850, Stem cell Technologies, Cambridge, MA, USA) on Matrigel (35 μg/cm) until the diameter of ESC colonies became 1-2 mm. To initiate the differentiation process, a similar method was followed to that in previous papers [37,38]. Briefly, on day 1, mTeSR1 medium was replaced by induction medium to produce definitive endoderm. The induction medium contained RPMI 1640 (#11875093, Gibco, Carlsbad, CA, USA) with 0.3% bovine serum albumin (#15561020, Gibco, Carlsbad, CA, USA), 1 × non-essential amino acids (#11140076, Gibco, Carlsbad, CA, USA), 2 mM glutamine (#A2916801, Gibco, Carlsbad, CA, USA) and 100 ng/mL Activin A (#CYT-569, Prospec Inc., East Brunswick, NJ, USA). On day 2, the induction medium was replaced with fresh induction medium containing 0.1 × insulin transferrin selenium complexes (ITS) (#I1884, Sigma, St. Louis, MO, USA) and on day 3 the culture medium was replaced with fresh induction medium containing 1 × ITS. Hepatocellular lineages were induced on day 4 with HCM containing 20 ng/mL BMP4 (#CYT-361, Prospec Inc., East Brunswick, NJ, USA) and 10 ng/mL FGF2 (#FGF2, R&D Systems, Minneapolis, MN, USA) for 5 days. On day 9, hepatoblasts formed and were switched to HCM containing 20 ng/mL HGF (#100-39, Peprotech Inc., Cranbury, NJ, USA) and cultured for 5 days to generate hepatocyte-like cells. After that, hepatocyte-like cells were cultured for another 5 days in HCM containing 10 ng/mL oncostatin M (OSM) (#CYT-231, Prospec Inc., East Brunswick, NJ, USA) and 0.1 μM dexamethasone (#D-085, Sigma, St. Louis, MO, USA) until harvest of the mature hepatocyte-like cells for analysis.

### 4.3. RNA Extraction and Reverse Transcription

Regarding the RNA isolation procedure from human liver samples, ~50 mg of liver tissue, which was preserved in an RNA*later* Stabilization Solution (#AM7024, Invitrogen, Carlsbad, CA, USA), was used to perform the RNA isolation using the miRNeasy column purification kit (#217004, Qiagen, Germantown, MD, USA) with on-column DNase treatment using the RNase-Free DNase Set (#79254, Qiagen, Germantown, MD, USA) according to the manufacturer’s instructions. The homogenization of tissue in 700 μL QIAzol (miRNeasy kit) was performed with a Power Gen 35 Homogenizer (Fisher Scientific, Kennebunk, ME, USA) for 5–10 s at 75% of the maximum speed until tissue was fully disrupted and the tissue/QIAzol mixture was clarified. Nuclease-free water was used for the final elution of RNA from the purification column. Concentrations and O.D. 260/280 purity ratio were determined by a Nanodrop 8000 (ThermoFisher, Waltham, MA, USA). RNA integrity was assessed using a TapeStation 4200 RNA ScreenTape assay (Agilent Technologies, Santa Clara, CA, USA).

Extraction of total RNA from each cell sample was performed using the TRIzol reagent (#15596018, Invitrogen, Tewksbury, MA, USA) according to the manufacturer’s instructions. Subsequently, the extracted total RNA was subjected to reverse transcription using the iScript™ cDNA Synthesis Kit (#1708891, Bio-Rad, Carlsbad, CA, USA) following the manufacturer’s protocol.

### 4.4. Primer Design for PCR and Quantitative RT-qPCR

PCR primers were designed to specifically amplify the target transcripts. Forward primers were chosen from the first exon of each transcript, while reverse primers were selected from the last exon. The precise positions of the PCR primers for each alternative transcript are illustrated in Figure 1B and the sequence information is provided in Table 2.

For RT-qPCR analysis, primers were carefully selected to ensure high specificity and efficiency in amplifying the target transcripts. The schematic diagram illustrating the primer design is depicted in Figure 2A, and the sequence information is provided in Table 3. All primers used in this study were synthesized by Integrated DNA Technologies (Coralville, IA, USA).

### 4.5. PCR

PCR amplification was conducted using the specifically designed primer pairs. The PCR reaction mixture comprised the cDNA template, primers, dNTPs (#R0241, Thermo Scientific, Waltham, MA, USA), and DreamTaq DNA polymerase (#EP0713, Thermo Scientific, Waltham, MA, USA). The amplification reactions were performed following the manufacturer’s instructions.

### 4.6. Gel Electrophoresis

Amplified PCR products were resolved by gel electrophoresis on 2% agarose gels (#IB70051, IBI Scientific, Dubuque, IA, USA). Subsequently, the gels were stained with ethidium bromide (#E1510, Sigma-Aldrich, Burlington, MA, USA) for 30 min. The DNA bands were visualized using ChemiDoc™ MP (Bio-Rad, Carlsbad, CA, USA), and their sizes were estimated by comparison to a 100 bp ladder (#SM0243, Thermo Scientific, Waltham, MA, USA).

### 4.7. Purification of PCR Products and Sanger Sequence

PCR products were subjected to purification from the gel using the QIAquick Gel Extraction Kit (#28704, QIAGEN, Germantown, MD, USA) according to the manufacturer’s instructions. The purified DNA samples were subsequently sent to Eurofins Scientific (Boston, MA, USA) for Sanger sequencing, aiming to determine the nucleotide sequence of the amplified fragments.

### 4.8. RT-qPCR

The RT-qPCR reactions were performed using iTaq™ Universal SYBR^®^ Green Supermix (#1725121, Bio-Rad, Carlsbad, CA, USA) according to the manufacturer’s instructions. The annealing temperature was set to 60 °C. β-actin was employed as the internal control for RT-qPCR normalization.

### 4.9. Data Analysis

The data are presented as means ± standard deviation (S.D.). Statistical analyses for data were performed using GraphPad Prism (La Jolla, CA, USA). Statistically significant differences in gene expression between two groups were assessed by a *t*-test. Significant *p*-values are indicated with asterisks as follows: * *p* < 0.05, ** *p* < 0.01, *** *p* < 0.001, **** *p* < 0.0001. Pearson correlation was used for the correlation analyses.

## Figures and Tables

**Table 1:** Information of the HNF1A-AS1 alternative transcripts in the genome browser.

Transcript ID *	Name	Length (bp)	Exon Number
ENST00000619441.1	HNF1A-AS1-205	343	2
ENST00000647473.1	HNF1A-AS1-207	1144	5
ENST00000701967.1	HNF1A-AS1-209	357	2
ENST00000701238.1	HNF1A-AS1-208	582	2
ENST00000646404.1	HNF1A-AS1-206	557	3
ENST00000537361.1	HNF1A-AS1-203	659	2
ENST00000539163.1	HNF1A-AS1-204	2455	1
ENST00000433033.3	HNF1A-AS1-201	718	4
ENST00000535301.2	HNF1A-AS1-202	546	2

Note: * The notation of these transcripts is based on the location of their last exon in the antisense strand of the genome.

**Table 2:** PCR primer sequences for validation of the alternative transcripts of HNF1A-AS1.

Transcript	Primer (Forward) 5′–3′	Primer (Reverse) 5′–3′	Amplicon Size (bp)
HNF1A-AS1-205	AGGAAGCACTTTGACCTCTG *	CCTACCCCACAGAGTCTGAT	300
HNF1A-AS1-207	ATCCCACCAAAGGGGGCT	TCACAGAAAAGAATCTGTTC	731
HNF1A-AS1-209	ATACAGGAAAGGGGAGCAGC **	TCCACATCAGGTCCCATG ***	220
HNF1A-AS1-208	AGGAAGCACTTTGACCTCTG *	TCCACATCAGGTCCCATG ***	280
HNF1A-AS1-206	ATACAGGAAAGGGGAGCAGC **	TCCACATCAGGTCCCATG ***	387
HNF1A-AS1-203	AGGAAGCACTTTGACCTCTG *	TGTTACAAGGTTCAGGGCTC	477
HNF1A-AS1-204	AGTTCCCTCCATCTAACATTCA	TTGTCTGGACTGAAGGACAA	321
HNF1A-AS1-201	GCCTGTGGCCATACAGGA	GACAGGAGCAAAACTGCT	501
HNF1A-AS1-202	AGGAAGCACTTTGACCTCTG *	ACAGAAGGAGACCCTGTC	298

Note: *, **, *** indicates that transcripts share the same primers.

**Table 3:** RT-qPCR primer sequences.

Transcript	Primer (Forward) 5′–3′	Primer (Reverse) 5′–3′	Amplicon Size (bp)
HNF1A-AS1-205N	ATGCTGTTTGGGCTGGTC	TGGGATTGGGTTCCTTTG	138
HNF1A-AS1-205	CACTCAGCCAGGATGAGG	TTTCCCTACCCCACAGAG	207
HNF1A-AS1-208	AGGAAGCACTTTGACCTCTG	TCCACATCAGGTCCCATG	280
HNF1A-AS1-203N	TAAACACTAGCCAACACCC	AGCAGCAGCTTGACAAAT	104
HNF1A-AS1-203	ACTCAGCCAGCTGCTCCCTCTA	GCTGTGGGTGGCAGAAGAGGAC	109
HNF1A-AS1-204	AGTTCCCTCCATCTAACATTCA	TTGTCTGGACTGAAGGACAA	321
CYP3A4	GATTGACTCTCAGAATTCAAAAGAAACTGA	GGTGAGTGGCCAGTTCATACATAATG	148
β-actin	GGACTTCGAGCAAGAGATGG	AGCACTGTGTTGGCGTACAG	234

## Data Availability

All raw data and processed data are stored on the OneDive of Zhong laboratory at the University of Connecticut. The data can be made available to the public upon request. When published, all raw data and processed data will also be deposited into an NIGMS dedicated repository followed by the NIGMS Data Management and Sharing Plan policy.

## Supplementary Materials

The following supporting information can be downloaded at: https://www.mdpi.com/article/10.3390/ncrna10020028/s1, Supplemental Table S1. Sequence.information of 6 confirmed transcripts.
