# Supplementary material for: Identification and Functional Characterization of Alternative Transcripts of LncRNA HNF1A-AS1 and Their Impacts on Cell Growth, Differentiation, Liver Diseases, and in Response to Drug Induction
Source: Noncoding RNA. 2024 Apr 21;10(2):28. doi: 10.3390/ncrna10020028 (PMC11053763; doi:10.3390/ncrna10020028)
Supplement: Supplementary file 1 [file ncrna-10-00028-s001.zip › ncrna-2931663-supplementary.pdf]

Supplemental Table S1. Sequence.information of 6 confirmed transcripts

Name	Sequence	Product Size (bp)
HNF1A-AS1-205N	CCAGGUCUCUGCUGGUCCCUAGGAAGCACUUUGACCUCUGCUUCCCCUUCA<br>AAUGCUGUGGAUCAAGCCUCCCCAACCCCACCAUCCCACCAAAGGGGGCUG<br>CUGACCGAGAGAGCUCACUCAGCCAGAGAUGACGUAUUGCUAUGCUGUUUG<br>GGCUGGUCUUUGAACUCCUGGCCUCAAGCAAUCCUCCUACCUCGGCCUCCC<br>AAUGUGCUGGGAUUACAGGAUGAGGGCAAAAGAGUUUAAGAUGGUGGAGC<br>CUUAUUAUGCAAAGGAACCCAAUCCCAGCGUCCACUGGAAGAAGACUGCCC<br>AGGACUUCUACCUUCCCGGGAACAUCUGCCCAGAGUCCACCAGCUGGAAGA<br>AGACUGCCCAGGACUUCUACCUUCCUGGGAACAUCUGCAUCAGACUCUGUG<br>GGGUAGGGAAAUAAACCUUCAUUGUGUUCA	437
HNF1A-AS1-205	CCAGGUCUCUGCUGGUCCCUAGGAAGCACUUUGACCUCUGCUUCCCCUUCA<br>AAUGCUGUGGAUCAAGCCUCCCCAACCCCACCAUCCCACCAAAGGGGGCUG<br>CUGACCGAGAGAGCUCACUCAGCCAGGAUGAGGGCAAAAGAGUUUAAGAUG<br>GUGGAGCCUUAUUAUGCAAAGGAACCCAAUCCCAGCGUCCACUGGAAGAAG<br>ACUGCCCAGGACUUCUACCUUCCCGGGAACAUCUGCCCAGAGUCCACCAGC<br>UGGAAGAAGACUGCCCAGGACUUCUACCUUCCUGGGAACAUCUGCAUCAGA<br>CUCUGUGGGGUAGGGAAAUAAACCUUCAUUGUGUUCA	343
HNF1A-AS1-208	AGCAUUAAAGAAAACCCAAUCGGGGCAGGCGGGAAGCACUCCAGCCUCCUU<br>GCAAGCUUGGGGCGCCUCAUUCUCUCCCGUGCCCCUUCUCCCAGCUGGGUCU<br>GAGCCCCCGCCCCCCUGGCUCCUCCGUUCUGCUUCUCCCAGCUUCUCUGUCC<br>AUCUCCCCCAAUGGCCUCUCUCUACACUCUCUGCCUCAGCUUCCAGGUCUCU<br>GCUGGUCCCUAGGAAGCACUUUGACCUCUGCUUCCCCUUCAAAUGCUGUGG<br>AUCAAGCCUCCCCAACCCCACCAUCCCACCAAAGGGGGCUGCUGACCGAGAG<br>AGCUCACUCAGCCAGAUAUCCCACCGAUGGCCAAGUGCCAGUUAGUCACGG<br>CCUCAGUGCAGCACCUCAUCCCUCCCAGAAAGCCAUGGCUCUGGACUCCACC<br>CACUGAAAACAGGGCUUUUGUGGGAAUACAAUGGGACAAGAAGAAAAACA<br>UGCUGAACCUGAUCUCCAUGGGACCUGAUGUGGAGUCACGUUCAUUCACAU<br>CACUGGCUUGCAGCCUCUCCACUCACAAGUUAUUGAUAAUCGAAAAAAUAA<br>AACACCAUGCGAUCGUA	582
HNF1A-AS1-203	GCGCCTCATTCTCTCCCGTGCCCCTTCTCCCAGCTGGGTCTGAGCCCCCGCCCCC<br>CTGGCTCCTCCGTTCTGCTTCTCCCAGCTTCTCTGTCCATCTCCCCCAATGGCCTC<br>TCTCTACACTCTCTGCCTCAGCTTCCAGGTCTCTGCTGGTCCCTAGGAAGCACTT<br>TGACCTCTGCTTCCCCTTCAAATGCTGTGGATCAAGCCTCCCCAACCCCACCAT<br>CCCACCAAAGGGGGCTGCTGACCGAGAGAGCTCACTCAGCCAGCUGCUCCCU<br>CUACCUGGAAACCUCCAUUUGUCAAGCUGCUGCUGUCUUCAAGAUCUACCU<br>GGUCAGAAUCUCCUGCUGUCCUCUUCUGCCACCCACAGCAAUGUGAGCCUU<br>CACUGGCUGGCCUUCCCUACUCACUGGAGCAGAGCUACGGCAUCAGAAUGC<br>CCUGGAGCAGGGGUUAGAAACACAGAUUUCUGAGUCUACACUCGAAGAUUU<br>UUUUUUCUUUCUUUCUUUUUUUUUGUUUUCCAAUUUUUCUAAUUUUAGAG<br>AUGGGGUCUCACCACGUUGCCCAGGCUGGUCUCAAGCUCCUGGACUCAGGU<br>CAUCCUACCACCUAGGCCUUCUAAAGUGUUAGGAUGAGCCCUGAACCUUGU<br>AACAUGAACAUUUAUUAGUUAUAUCUGGAAA	659
HNF1A-AS1-203N	GCGCCTCATTCTCTCCCGTGCCCCTTCTCCCAGCTGGGTCTGAGCCCCCGCCCCC<br>CTGGCTCCTCCGTTCTGCTTCTCCCAGCTTCTCTGTCCATCTCCCCCAATGGCCTC<br>TCTCTACACTCTCTGCCTCAGCTTCCAGGTCTCTGCTGGTCCCTAGGAAGCACTT<br>TGACCTCTGCTTCCCCTTCAAATGCTGTGGATCAAGCCTCCCCAACCCCACCAT<br>CCCACCAAAGGGGGCTGCTGACCGAGAGAGCTCACTCAGCCAGAGAGCCAGC<br>UGUUAAACACUAGCCAACACCCCAUUGAUGCUGUUCCUUCUACCUGAAACA<br>CCUCGCAUUGCCGCUGCUCCCUCUACCUGGAAACCUCCAUUUGUCAAGCUG<br>CUGCUGUCUUCAAGAUCUACCUGGUCAGAAUCUCCUGCUGUCCUCUUCUGC<br>CACCCACAGCAAUGUGAGCCUUCACUGGCUGGCCUUCCCUACUCACUGGAG<br>CAGAGCUACGGCAUCAGAAUGCCCUGGAGCAGGGGUUAGAAACACAGAUUU<br>CUGAGUCUACACUCGAAGAUUUUUUUUUCUUUCUUUCUUUUUUUUUGUUU<br>UCCAAUUUUUCUAAUUUUAGAGAUGGGGUCUCACCACGUUGCCCAGGCUGG	732

	UCUCAAGCUCCUGGACUCAGGUCAUCCUACCACCUAGGCCUUCUAAAGUGU<br>UAGGAUGAGCCCUGAACCUUGUAACAUGAACAUUUAUUAGUUAUAUCUGG<br>AAA	
HNF1A-AS1-204	AGUAAGUGCAGUAGGUAGGCCAAGAUAACUAUUAAGUAUGUCCUAACAAG<br>GUAGCUGCUUGAAAAAAAUAAUGCACAUAAAUUAAAAAAAAAAAAACUAU<br>UUUCACUCAUUCUUAACUUCUCGUAUGGAUGUACUAACGGAGGCCUGUGC<br>CUGUUGGGCUGUCCACAGCUACUCAAACCUUGAGAUUAGAUUGGACGCUAU<br>CUAAUCUCAUUUCCUGUUACACAAUAACUUCUGCAUGGCACUUGCUUUUAA<br>UCAUGACAACUGCCAUACACUCAGCUUUACAAAAUCAUGUCAUUGUGGAAA<br>GGAAGUUCCCUCCAUCUAACAUUCAAAUUUAACUAUCCUGAGCUGGUAGUU<br>UGCAAGGUGUCCAACAGAUGUCACUGUGUUUCCUUCGCAAAUUCUCUAAAA<br>UAGUCUGCUGUGUCCCGGGGCAUAGUAGAUAUUCAAGAAAUGGUGGCUAU<br>GAGUUUUAUCACAAUUCUCUCCUACUGGGUGAGCAGCUGUUUGCAAGACUA<br>AAAUUCGGGCGAGGCACAGGCUUCCCCUUGGUCUUAGCAGCUAGCACAAGA<br>CGAGUGUCCCUUCAGCCAGUCUCAGAGCUCUUUGGCAAUUCAACUGACCCU<br>UGUCCUUCAGUCCAGACAAUGCCCCAGGGCCCACCUGCAUUCAAACUCGGA<br>CUGUUCUCCUUCCCACCCCUAUUAUCUUUUCAACUUUAUGGACAGUACAUG<br>CAAUUUUCUCAUUCAUUUUCUCAUUUAACAUCUAACAGCUUCAUGAUAUCC<br>CCAUUUUACAGAUAAGGAAACCAAGGCUUUAAGAGGAUAGGGACCCUAAU<br>CGGCUAAGGCAGAAUUAGGAUUUGAACCCAGAUCCCCAUGACGCCAGAAUC<br>UAAGCCUUGACCAAAUAUGUGUUACUAUUACUUUUGUUGUUGUUGUUGUU<br>CUGGGUAACUCCAACCCUCUGCUCGUUGGCCUUGUCACCUCAGUGCUGAUU<br>UUGCAGAUGAAGAGACUAAGGUUCCGAGGUGCUAGAGCACAGUGGUUAGG<br>AACACACACUCUAGAGCCAGAGUGCCUGGGUUUGAGCCUCGUUCUGCCGUU<br>UACUAGCCGUAUGGCCUUGGGCAACUUGCUUAACUUCUCUUUGCCUCAGUU<br>CCUCUUCUCUUGGGUUGUUAGGAGAAUGAAUGAGGUGAUAUCCACAGUGU<br>CCAACACCCUGCAAGCGGCCAGGUGUGGUGGAUCACACCUGCAAUCCCAGC<br>ACUUUGGGAGGCCGAGGCAGGUGGAUCAUUUGAGGUCAGGAGUUCGAGACC<br>AGUCUGGCCAACAUGGCGAAACCUCAUUUCUACUAAAAAUACAAAAAUUAG<br>CCGGGCGUGGUGGCAAACAACUGUAAUCCGAGCUACUAGGGAGGCUGAGGC<br>AGAAGAAUCGCUUGAACCCAGGAGACAGAGGUUGCAGUGAGCCAAGAUUGC<br>ACCACUGCACUCCAGCCUGGGCGACAGAGUGAGACCCUGCCUCAAAAACUA<br>AAAAUAAAAAAUGAACAGUGUCCAACACCUUGAAAGCAUUCCGCAUGACUG<br>AACGAUCACUGUUACGUGAUUACCAACAUGACGACCCCACUUCUCUUGGCC<br>AAACUGAGUUCUCUUCUCUUGUAAAAACUUCUGCAUAUGAAGUCACCCAUU<br>CCAUUUGACUCUUUAGCCCUCAUCCUGGCAUUCAAGGGCAUGGACGACUCA<br>ACCCCACUCUCUAGCCAGCCUGACCUCUCCAUUCCCUUCUCUGGCGUAGAGG<br>CCACUCCCACCACCUGCCCUUACCCCCUGCCCACUUUUGUCAGCCUGUACUG<br>UGUUGAAGCUCCCAGGAGAGACAACUGCAGUCAUCACUUUCCACUGUGUUC<br>AGUGAUGUCAGUUCGGCCCAGCUGGAGGUACUCACAUCAUGGAAACAGACC<br>AUCAGAUUUGGUGUGUGUGUGUUUUAUUUUGCUUUUUCAGAGAGCCAGCU<br>GUUAAACACUAGCCAACACCCCAUUGAUGCUGUUCCUUCUACCUGAAACAC<br>CUCGCAUUGCCGGUGAGCUCUAAAGACAAAAGGACCUGGGUCUGCAUUUCC<br>CAAAGGCAAGACUCUGGUAUCACCUGCAUAUUAUUUUCCCAUAUUUGAGCA<br>CCACUGCCUUUUGCAUUAUUUACUUAGCAGUUUUGCUCCUGUCAACUCACU<br>UUUUUAAAACUAAACUUAUUUUAAAAAGAAAAGUUUUGGCCGGGAGCGGU<br>GGCUCAUGCCUGUAAUCCCAGCACUUUGGGAGGCCGAGGUGGGCAGAUCAC<br>CUGAGGUCGGGAGUUCGAGACCAGCCUGACCAACAUGGAGAAACUCCGUCU<br>CUACUAAAAAUACAAAAUUAGCUGGGCGUGGUGGCGCAUGCCUGUAAUCCU<br>AGCUACUCGGGAGGCUGUGGCAGGAGAAUUGCUUGAACCCAGGAGGCGGGG<br>GUUGCAGUGAGUCGAGAUCGCCCCAUUGUACUCCAGCCUGGGGAACAAGAA<br>CGAAACUCCGUCUC	2455

Exons are labeled in colors that match Fig.2 A.
